# Reproducibility of Transcranial Doppler ultrasound in the middle cerebral artery

**DOI:** 10.1186/s12947-018-0133-z

**Published:** 2018-09-11

**Authors:** Jakub Kaczynski, Rachel Home, Karen Shields, Matthew Walters, William Whiteley, Joanna Wardlaw, David E. Newby

**Affiliations:** 1 British Heart Foundation Centre for Cardiovascular Science, University of Edinburgh, Chancellor’s Building, 49 Little France Crescent, Edinburgh, EH16 4SA UK; 2 College of Medicine and Veterinary Medicine, University of Edinburgh, 47 Little France Crescent, Edinburgh, EH16 4TJ UK; 3 Stroke Unit, Queen Elizabeth University Hospital, 1345 Govan Road, Glasgow, G51 4TF UK; 4 College of Medical, Veterinary and Life Sciences, Wolfson Medical School Building, University of Glasgow, University Avenue, Glasgow, G12 8QQ UK; 5 Royal Infirmary of Edinburgh, 51 Little France Crescent, Old Dalkeith Road, Edinburgh, EH16 4SA UK

**Keywords:** Transcranial Doppler, Microembolic signals, Carotid artery stenosis, Ischaemic stroke

## Abstract

**Background:**

Transcranial Doppler ultrasound remains the only imaging modality that is capable of real-time measurements of blood flow velocity and microembolic signals in the cerebral circulation. We here assessed the repeatability and reproducibility of transcranial Doppler ultrasound in healthy volunteers and patients with symptomatic carotid artery stenosis.

**Methods:**

Between March and August 2017, we recruited 20 healthy volunteers and 20 patients with symptomatic carotid artery stenosis. In a quiet temperature-controlled room, two 1-h transcranial Doppler measurements of blood flow velocities and microembolic signals were performed sequentially on the same day (within-day repeatability) and a third 7–14 days later (between-day reproducibility). Levels of agreement were assessed by interclass correlation co-efficient.

**Results:**

In healthy volunteers (31±9 years, 11 male), within-day repeatability of Doppler measurements were 0.880 (95% CI 0.726–0.950) for peak velocity, 0.867 (95% CI 0.700–0.945) for mean velocity, and 0.887 (95% CI 0.741–0.953) for end-diastolic velocity. Between-day reproducibility was similar but lower: 0.777 (95% CI 0.526–0.905), 0.795 (95% CI 0.558–0.913), and 0.674 (95% CI 0.349–0.856) respectively. In patients (72±11 years, 11 male), within-day repeatability of Doppler measurements were higher: 0.926 (95% CI 0.826–0.970) for peak velocity, 0.922 (95% CI 0.817–0.968) for mean velocity, and 0.868 (95% CI 0.701–0.945) for end-diastolic velocity. Similarly, between-day reproducibility revealed lower values: 0.800 (95% CI 0.567–0.915), 0.786 (95% CI 0.542–0.909), and 0.778 (95% CI 0.527–0.905) respectively. In both cohorts, the intra-observer Bland Altman analysis demonstrated acceptable mean measurement differences and limits of agreement between series of middle cerebral artery velocity measurements with very few outliers. In patients, the carotid stenoses were 30–40% (*n* = 9), 40–50% (*n* = 6), 50–70% (*n* = 3) and > 70% (*n* = 2).

No spontaneous embolisation was detected in either of the groups.

**Conclusions:**

Transcranial Doppler generates reproducible data regarding the middle cerebral artery velocities. However, larger studies are needed to validate its clinical applicability.

**Trial registration:**

ClinicalTrial.gov (ID NCT 03050567), retrospectively registered on 15/05/2017.

## Background

Ischaemic stroke remains a major global cause of disability and death that is associated with an enormous social and economic burden [1]. Up to 25% of ischaemic strokes are caused by atherosclerosis of the internal carotid artery [2, 3]. Carotid atherosclerosis is a complex disease that is characterised by the deposition of luminal atheroma that may rupture, thrombose and embolise [2]. The resulting thromboembolism can lead to a stroke or transient ischaemic attack (TIA) [4].

Transcranial Doppler is a well established real-time imaging modality that evaluates cerebral blood flow velocity and detects microembolic signals in patients who suffer from cerebral or retinal ischaemia [5]. Microembolic signals in symptomatic carotid artery stenosis are associated with an increased risk of a recurrent ipsilateral focal ischaemia [6–14] and and correlate with a greater number of magnetic resonance imaging detectable cerebral infarcts when compared with patients free from microembolism [15–18]. The intraoperative transcranial Doppler has enabled clinicians to lower the rate of the most serious post carotid endarterectomy complication such as thromboembolic stroke from 4 to 0.2% through detection of the middle cerebral artery flow cessation due to the intraluminal carotid artery thrombosis [19, 20]. Whereas, transcranial Doppler directed infusion of Dextran 40 has in some centres successfully erased the rate of postoperative thromboembolic cerebral ischaemia from 2.7 to 0% [8, 21]. Despite these benefits from transcranial Doppler, routine use has not been advocated amongst vascular specialists.

Although multiple studies have been conducted on flow velocities in basal cerebral arteries in both healthy volunteers and patients [22–33], reproducibility data are limited to a hand full of reports. These include four articles involving healthy subjects [34–37] and one study that recruited patients with clinical diagnosis of ischaemic stroke (*n* = 3) or TIA (*n* = 7) but provided no information regarding the clinical type of neurovascular event or underlying carotid artery stenosis [5]. In contrast, published data on microembolic signals detection in patients with symptomatic carotid artery stenosis includes systematic reviews, meta-analyses [38–41] international multicenter reproducibility studies that have described the reproducibility of transcranial Doppler as sufficient for clinical use [10, 42, 43].

Our objective was to assess the intra-observer repeatability and reproducibility of transcranial Doppler for velocimetry measurements and microemboli detection in healthy volunteers and patients with symptomatic carotid artery stenosis that could form the basis for our future study investigating reliable identification of a vulnerable carotid plaque.

## Methods

### Study design

This was an observational investigative study. The study was approved by the local Research Ethics Committee (16/SS/0217), and written consent was obtained from all participants. The research protocol is available on ClinicalTrial.gov (ID NCT 03050567).

### Study population

Cohorts (*n* = 20 per cohort) of healthy volunteers and patients with symptomatic carotid artery stenosis were recruited between March–August 2017. Among the patient group, five patients that were excluded due to an absent temporal window, were subsequently replaced. Healthy volunteers were > 18 years old and had no previous history of cerebrovascular disease. Patients with evidence of an acute neurovascular syndrome (stroke, TIA, retinal ischaemia) due to carotid artery disease were recruited from the acute neurovascular clinics at Edinburgh Royal Infirmary within a maximum of 14 days of symptom onset. The inclusion criteria were the symptomatic cerebrovascular event (stroke, TIA or amaurosis fugax) and radiological confirmation of carotid artery stenosis of > 30%. This included patients scheduled for carotid endarterectomy (> 50% for men and > 70% for women, by North American Symptomatic Carotid Endarterectomy Trial criteria) or treated conservatively with an optimal medical therapy (if patient declined surgical intervention or is outside surgical criteria for carotid endarterectomy) [3].

### Study protocol

**Fig. 1:**
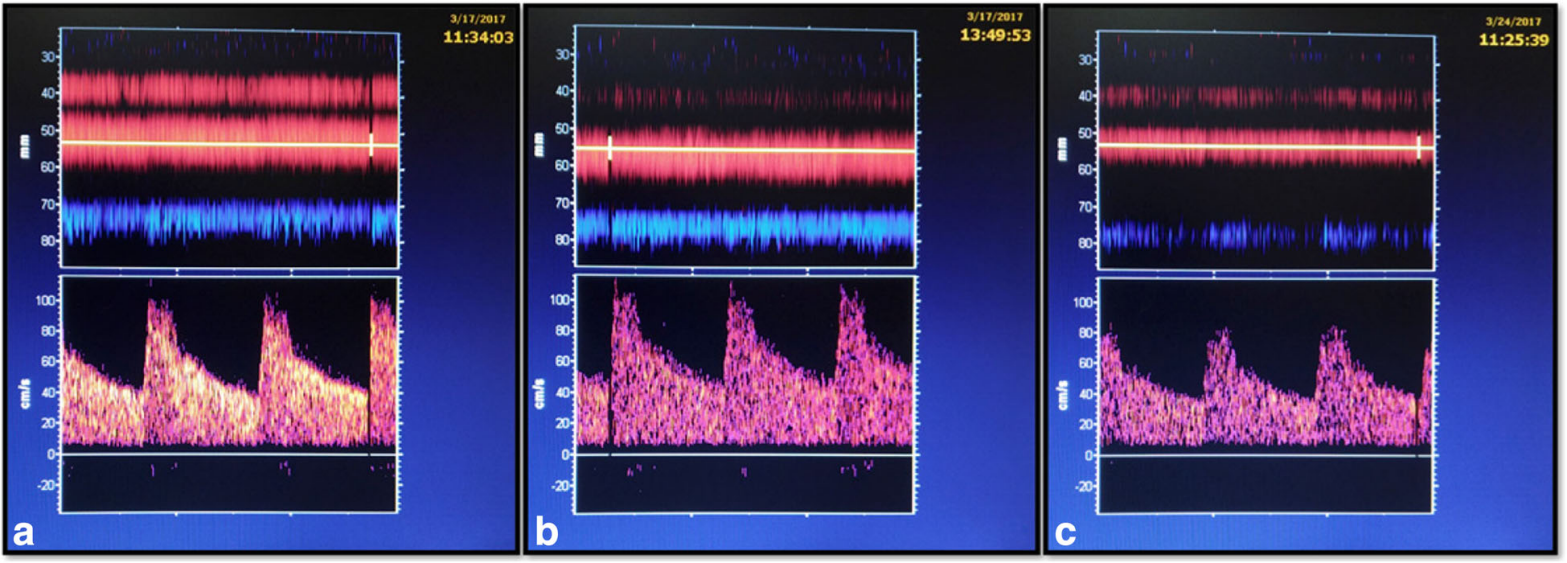
Three transcranial Doppler examinations of the same participant (visit 1: images **a** and **b**; visit 2: image **c**)

All subjects underwent clinical evaluation prior to participation. In the patient group, this included assessment of relevant carotid Doppler ultrasound and brain imaging investigations (computed tomography or magnetic resonance imaging). In both cohorts, three 1-h transcranial Doppler measurements were performed by the same examiner over two study visits. During the first study visit, two examinations were performed separated by 1 h (Fig. 1). The final (third) examination was obtained on a separate study visit within 14 days of the first examination (Fig. 1).

### Transcranial Doppler ultrasound

All examinations were performed in a semi-recumbent position in a quiet temperature-controlled room. The middle cerebral artery was identified through the temporal window and the sample volume adjusted to obtain a stable visually and acoustically optimal signal. In healthy subjects, the side of the middle cerebral artery insonation was randomly allocated. In patients with symptomatic carotid artery stenosis, transcranial Doppler was performed on the symptomatic middle cerebral artery (ipsilateral to the index event). A head frame (Marc 600 Spencer Technologies, USA) was fitted to reduce motion and to secure a constant angle of the middle cerebral artery insonation depth at 40–65 mm from the skull surface. All recordings were made using the ST^3^ Transcranial Doppler Ultrasound System (Spencer Technologies, USA) with a 2-MHz transducer. Emboli were identified using characteristic short audible sound (range 10–100 ms, intensity threshold above 7 dB) and spectral appearance using the International Consensus Group microembolus identification criteria and assisted by an automated Embolus Detection Software (Spencer Technologies, USA) [44]. The Doppler wave forms were reassessed to exclude artefact and confirm the presence of true emboli. The mean of maximal, mean and end-diastolic flow velocities were determined from the mean of measurements obtained over ten cardiac cycles.

### Statistical analysis

Continuous variables were expressed as mean ± standard deviation for normally distributed data, and categorical variables were expressed as total and percentage. To quantify intra-observer repeatability and reproducibility of imaging measurements, the intra-class correlation co-efficient (ICC) was calculated and Bland-Altman analysis undertaken. Statistical significance was taken as a two-sided *P* < 0.05. Statistical analyses were performed with the use of IBM SPSS Statistics for Mac, version 23 (Armonk, New York, IBM Corp, USA).

## Results

All participants tolerated transcranial Doppler examinations well and completed all assessments.

### Healthy volunteers

In total, 60 transcranial Doppler assessments were performed on 20 healthy volunteers who had a mean age of 31±9 years, and 11 were male. All subjects had the temporal window available, and the mean middle cerebral artery insonation depth was 51 mm (Table 1) with peak velocities averaging around 70–85 cm/s (Table 1).

**Table 1:** Summary of the middle cerebral artery blood flow velocity and insonation depth in healthy volunteers and patients

Healthy Volunteers			
Velocity (cm/s)	Examination 1	Examination 2	Examination 3
Peak	75.70 ± 23.91	72.00 ± 20.28	82.75 ± 19.93
Mean	49.95 ± 15.30	47.05 ± 12.85	52.60 ± 11.73
Diastolic	34.70 ± 11.28	29.50 ± 10.65	35.45 ± 8.40
MCA depth (mm)	51.75 ± 2.65	51.75 ± 2.94	51.20 ± 2.93
Patients			
Velocity (cm/s)	Examination 1	Examination 2	Examination 3
Peak	73.70 ± 18.94	73.10 ± 16.62	71.20 ± 17.62
Mean	45.40 ± 11.79	45.20 ± 10.83	43.50 ± 9.96
Diastolic	27.30 ± 8.90	27.10 ± 8.14	25.60 ± 6.57
MCA depth (mm)	51.40 ± 5.33	51.55 ± 5.21	51.65 ± 5.24

Data are presented as mean ± standard deviation. MCA, middle cerebral artery

**Fig. 2:**
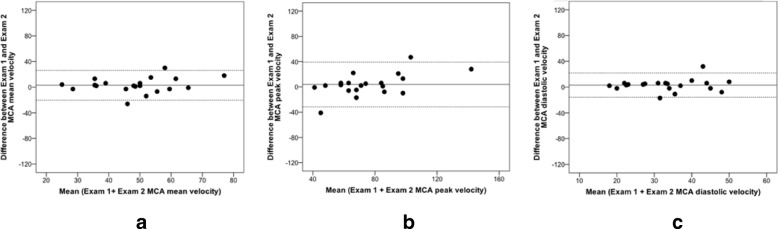
Repeatability in healthy volunteers (Examination 1 vs Examination 2): Bland Altman analysis for middle cerebral artery velocity. **a**) Peak. **b**) Mean. **c**) End-diastolic

**Fig. 3:**
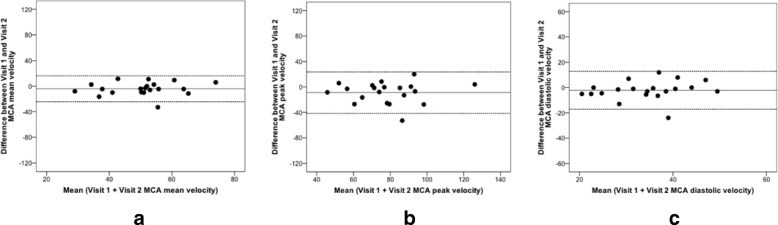
Reproducibility in healthy volunteers (visit 1 vs Visit 2): Bland Altman analysis for middle cerebral artery velocity. **a**) Peak. **b**) Mean. **c**) End-diastolic

Overall, the ICC for both repeatability and reproducibility in healthy volunteers group revealed a good reliability (ICC 0.75–0.90) with wider confidence intervals obtained for the peak and mean reproducibility values when compared with the repeatability measurements (Table 2). An intra-observer Bland Altman analysis demonstrated acceptable mean measurement differences and limits of agreement between series of middle cerebral artery velocity measurements with very few outliers (Figs. 2 and 3). As expected no microembolic signals were detected.

**Table 2:** Summary of ICC velocity values for repeatability and reproducibility assessments in healthy volunteers and patients

Healthy Volunteers		
Repeatability (Exam 1 vs Exam 2)	ICC	95% CI
Peak	0.880	0.726–0.950
Mean	0.867	0.700–0.945
End-diastolic	0.887	0.741–0.953
Reproducibility (Visit 1 vs Visit 2)	ICC	95% CI
Peak	0.777	0.526–0.905
Mean	0.795	0.558–0.913
End-diastolic	0.674	0.349–0.856
Patients		
Repeatability (Exam 1 vs Exam 2)	ICC	95% CI
Peak	0.926	0.826–0.970
Mean	0.922	0.817–0.968
End-diastolic	0.868	0.701–0.945
Reproducibility (Visit 1 vs Visit 2)	ICC	95% CI
Peak	0.800	0.567–0.915
Mean	0.786	0.542–0.909
End-diastolic	0.778	0.527–0.905

Date are reported as mean with 95% limits of agreement for assessments

### Patients

**Fig. 4:**
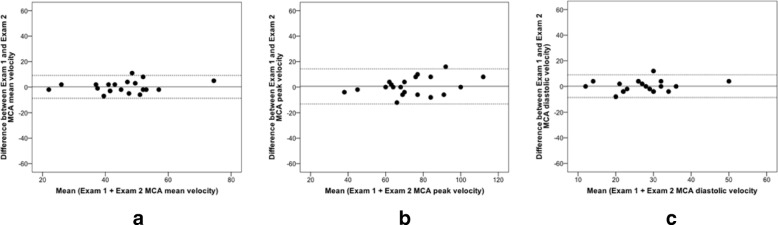
Repeatability in patients (Examination 1 vs Examination 2): Bland Altman analysis for middle cerebral artery velocity. **a**) Peak. **b**) Mean. **c**) End-diastolic

**Fig. 5:**
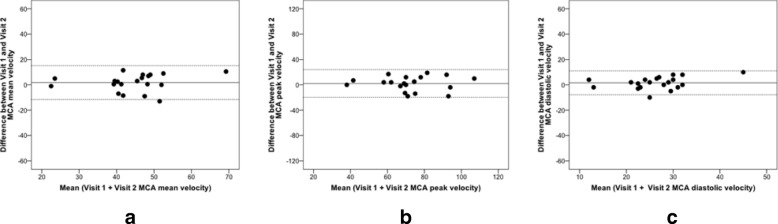
Reproducibility in patients (visit 1 vs Visit 2): Bland Altman analysis for middle cerebral artery velocity. **a**) Peak. **b**) Mean. **c**) End-diastolic

Patients had a mean age of 72±11 years, and 11 were men (Table 3). Presenting diagnosis included 18 transient ischaemic attacks (11 cerebral, 7 ocular) and 2 cases of ischaemic stroke. The degree of stenoses measured by Duplex ultrasound scan were: 30–40% (*n* = 9), 40–50% (*n* = 6), 50–70% (*n* = 3) and > 70% (*n* = 2). Five patients (4 females, 1 male) had an absent acoustic temporal window. The mean middle cerebral artery insonation depth was 51 mm (Table 1) and peak cerebral artery blood flow velocities were 70–75 cm/s (Table 1). The overall intra-observer ICC for repeatability and reproducibility displayed at least good (ICC 0.75–0.90) agreement that reached an excellent agreement (ICC > 0.90) for the peak and mean repeatability velocity values (Table 2). Similarly, wider confidence intervals were found for the peak and mean reproducibility values when compared with the repeatability assessments. The Bland Altman plots showed acceptable mean measurement differences and limits of agreement between series of middle cerebral artery velocity measurements with very few outliers (Figs. 4 and 5). No microembolic signals were detected during transcranial Doppler assessments.

**Table 3:** Baseline characteristics of patients

Characteristics	*n* (%) Total = 20
Demographics	
Age, years	72±10.6
Male	11 (55)
Vital signs	
Systolic BP	146.6±27.3
Diastolic BP	77.4±11.0
Heart rate/min	70.15±14.89
Smoker status	
Current smoker	2 (10)
Former smoker	12 (60)
Never smoked	6 (30)
Presenting diagnosis	
Cerebral TIA	9 (45)
Ocular TIA	7 (35)
Stroke	2 (10)
Duplex scan severity of stenosis (%)	
30–40%	9 (45)
4–50%	6 (30)
50–70%	3 (15)
> 70%	2 (10)
Duration between index event and 1st transcranial Doppler (days)	11.6±2.4
Relevant medical history	
Hypertension	15 (75)
Hypercholesterolemia	20 (100)
Diabetes Mellitus	3 (15)
Ischaemic Heart Disease	4 (20)
Peripheral Vascular Disease	1 (5)
TIA	4 (20)
Stroke	3 (15)
Chronic Obstructive Pulmonary Disease	3 (15)
Baseline medication therapy	
Antiplatelet	18 (90)
Anticoagulant	2 (10)
Statin	20 (100)
Beta-blocker	4 (20)
Angiotensin-converting enzyme inhibitor	7 (35)

Data are presented as mean (standard deviation) or as percentage (%) where appropriate

## Discussion

In this study, we have demonstrated that transcranial Doppler generates reproducible data regarding the velocity measurements. Transcranial Doppler utilises an acoustic temporal bone window through which the ultrasound beam can focus on the middle cerebral artery, which receives 80% of an ipsilateral internal carotid artery inflow [27]. The obtained middle cerebral artery insonation depth in both cohorts reflects published data [10, 45, 46].

In general, the success of transcranial Doppler imaging diminishes with an older age due to an increased temporal bone thickness that impairs the transmission of ultrasound waves through the skull [47, 48]. This has been observed primarily in approximately 10% of non-Caucasian elderly female participants [49]. However, others report temporal window failure in almost third of examined subjects [50].

Multiple studies described substantially different normal reference velocity values of cerebral arteries blood flow [22, 24, 25, 27, 29, 31] but the most frequently quoted normal middle cerebral artery velocity under resting condition ranges from 35 to 90 cm/sec with a mean of 60 cm/sec [29]. Our velocity values mirror the results published by others, except for the lower mean diastolic middle cerebral artery velocity. However, this could be explained by various physiological and technical factors that can affect velocity readings. First, physiological cardiovascular changes such as heart rate, blood pressure, respiratory rate, arterial carbon dioxide tension alter middle cerebral artery blood flow on a daily basis [51, 52]. Second, psychological factors (emotional state, fatigue) by influencing the above physiological cardiovascular autonomic responses can impact on the cerebral blood flow [51]. Unsurprisingly, changes in the cerebral metabolism due to cognitive activation also affect the middle cerebral artery blood flow. Some authors demonstrated that arithmetic activity produced very similar values to the resting blood flow values, whereas higher levels of arithmetic difficulty produced smaller changes in the blood flow [53]. Therefore, the above factors could have potentially influenced the obtained velocity values.

The main technical aspect that can impact on velocity measurement is the angle of insonation that is obtained between the middle cerebral artery and ultrasound beam [5, 34]. However, this is more relevant when large acoustic window such as the foramen of magnum is used, because it permits significant angle variation [34]. Fortunately, small temporal window with a sharp angle of insonation (0°-30°) that is relatively stable minimises any influence on obtained velocities values [5]. Hence, the maximum error has been estimated to be less than 15% [34, 35]. Finally, individual variability of the middle cerebral artery size, length and tortuosity are also contributing to the scattering of the velocity measurements [27, 29, 37].

In general, cerebral flow velocity decreases with age in a bimodal pattern with a first decline above the age of 40 years and a further reduction above 60 years of age [22, 26, 27, 54]. Unsurprisingly, our data demonstrate similar results with lower velocity values in patients cohort when compared with the healthy volunteers. Overall the obtained ICC values in our study represent a good repeatability and reproducibility in both cohorts. However, the peak and mean ICC repeatability values recorded in patients group reached an excellent agreement (ICC > 0.90). In both cohorts, the peak and mean ICC reproducibility values decrease with wider confidence intervals when compared to the repeatability values. This likely reflects the combination of technical variation and biological variation which will be much greater when measurements are conducted on separate days rather than within a day. For example, this could include probe displacement from the original middle cerebral artery segment that was sampled during the first study visit. This may also reflect the well described anatomical variability of the circle of Willis including diameter discrepancy of the individual parts of the middle cerebral artery [27, 29]. In effect, an over or under-estimated velocity values can be reported depending on the diameter of an insonated artery. The slightly higher ICC values obtained in the patients group could be explained by the lower range of physiological fluctuations and more consistent velocity measurements [28].

Finally, the equipment characterists such as head frame that supports the transducers could account for some differnces in velocity values. In our study we have used a professional head frame system (Marc 600 Spencer Technologies, USA) that minimises the motion and maintains a constant angle of insonation of the middle cerebral artery. Interestingly, no single reproducibility study on velocity measurements described any form of secure fixation of transducers during the examinations [5, 34–37]. Similarly, systematic reviews and meta-analyses on microembolic signals detection provide no information on any head-frame systems used by individual studies [38–41]. This raises many questions regarding the methodological aspects of these studies that have been conducted more than 20 years ago.

Although our data regarding transcranial Doppler velocities measurements echoes other researchers findings, it should be interpreted with caution owing to many methodological limitations of the published analyses including a limited number of reproducibility studies that contain small sample size and variable imaging protocols. Furthermore, evidence for the transcranial Doppler criteria to predict the degree of intracranial arteries stenoses remains inconclusive and controversial [18]. Several studies failed to demonstrate reproducible data on specific cut-off points for the velocities values with the percentage of stenosis [30, 32, 33, 55–57]. Some authors have proposed middle cerebral artery velocity of > 80 cm/sec as a criterion for stenosis [57], whereas others used velocities > 100 cm/sec when diagnosing stenosing lesions [54]. In contrast, some researchers have highlighted the importance of additional measurements such as side-to-side differences in velocities (> 30%) or increase in velocity (> 50%) along with the assessment of collateral flow using temporary manual occlusions of the common carotid artery [30, 32]. However, one must remember potential pitfalls with such approach because high velocities in collateral circulation can indicate different diameters of the middle cerebral arteries on two sides [30]. In effect, high blood flow velocities may be caused simply by the smaller diameter of MCA despite otherwise normal anatomy [33]. Finally, the largest study (The Stroke Outcomes and Neuroimaging of Intracranial Atherosclerosis (SONIA) Trial) that attempted to validate transcranial Doppler findings with magnetic resonance angiography against the standard cerebral digital subtraction angiography regarding the identification of intracranial arterial disease revealed disappointingly low results of positive predictive values for transcranial Doppler (36%) and magnetic resonance angiography (59%) [55]. In effect, the transcranial Doppler’s clinical applicability regarding the abnormal velocity values assessment remains limited.

Despite complete 1-h transcranial Doppler assessments performed in our study, the lack of microembolic signals in patients with symptomatic carotid artery stenosis was disappointing. The reported incidence varied from 12 to 100% in individual studies [40, 58, 59]. Nevertheless, considerable differences regarding criteria for microembolic signals detection, timing after stroke, duration of monitoring and antithrombotic agents used have been identified among many studies [10, 40, 58]. Consequently, the majority of published data described microembolic signals in about 30–40% of individuals with symptomatic carotid artery stenosis when transcranial Doppler was performed for 1 h [39, 40, 59, 60].

Still, there are several potential explanations for the absent embolisation. Thromboembolism is a dynamic and random process with a generally reported low frequency of microembolic signals during 1-h long examination [60, 61]. Although 1-h long transcranial Doppler evaluation time is recommended for patients with symptomatic carotid artery stenosis, longer assessments increase the chances of successful emboli detection [60]. This was demonstrated by ambulatory recordings (greater than 5 h) with portable transcranial Doppler equipment that has yield greater number of microembolic signals when compared with the traditional 60 min approach [62, 63]. However, at present, an ambulatory transcranial Doppler recording remains primarily a research tool due to lack of a robust equipment.

Another possible explanation refers to the severity of carotid artery stenosis and plaque morphology. Microembolic signals are more common in patients with the higher degree of carotid artery stenosis, which in turn is associated with specific carotid plaque features reported histologically such as ulceration, intraplaque haemorrhage and surface thrombus [11, 18, 39, 49, 59, 64, 65]. These high-risk plaque features are more likely to lead to the development of stroke because they produce larger emboli that consist of thrombi [59]. Whereas, small embolic particles comprising of fibrin and platelets aggregates that lodge in small arteriolar branches, may be lysed by endogenous protective haemostatic defences, hence clinically may represent TIA [59]. The majority of our patients had a non-surgical grade of carotid artery stenosis and presented with TIA. Therefore, these factors could be potentially responsible for no detectable microembolic signals.

The various components of microembolic signals responds differently to treatment [39]. For example, antiplatelet agents are more effective for emboli originating from the symptomatic carotid artery stenosis, and reduce the rate of microembolic signals [11, 12]. On the other hand, anticoagulants deal more effectively with microembolic signals from a cardiac source [11, 39]. The majority of participants (90%) in our study have been on an antiplatelet agent at the time of the first transcranial Doppler assessment, and this could represent another potential confounder. Finally, microembolic signals are more likely to be detected within the first week after the index event, and in patients with recent stroke rather than with TIA [66]. Again, we have performed transcranial Doppler as soon as possible, but due to various logistic factors, only two patients had transcranial Doppler within seven days from their index event.

The main limitations of this study are the small sample size, and single-centre design. However, the main purpose of the study was to demonstrate reproducibility of the transcranial Doppler and this was achieved. At present, transcranial Doppler remains underutilised in clinical practice due to lack of human expertise, time-consuming recordings with the need for a continuous visual and audible evaluation [13, 60]. Furthermore, unsolved technical and methodological limitations of transcranial Doppler regarding the velocity assessments restrict its clinical applicability. However, its use during carotid surgery has shown that the clinical use of this non-invasive, non-ionising, portable and safe technique could be extended to vascular surgery specialists as part of the routine perioperative strategy that could reduce the risk of neurovascular events even further [20].

## Conclusions

Our findings indicate that transcranial Doppler provides reproducible data on middle cerebral artery velocities. However, these findings should be interpreted with caution for the many technical and methodological limitations that the transcranial Doppler still presents. Larger studies with the colour transcranial Doppler may enable delivery of a robust data on velocity assessments along with the quantification of intracranial stenoses.

## Abbreviations

ICC: Intra-Class Correlation
SD: Standard Deviation
TIA: Transient Ischaemic Attack
